# *Varicus lacerta*, a new species of goby (Teleostei, Gobiidae, Gobiosomatini, *Nes* subgroup) from a mesophotic reef in the southern Caribbean

**DOI:** 10.3897/zookeys.596.8217

**Published:** 2016-06-08

**Authors:** Luke Tornabene, D. Ross Robertson, Carole C. Baldwin

**Affiliations:** 1 Division of Fishes, Department of Vertebrate Zoology, National Museum of Natural History, Smithsonian Institution, PO Box 37012, Washington, DC 20013-7012, U.S.A.; 2 Smithsonian Tropical Research Institute, Balboa, Republic of Panama

**Keywords:** Systematics, molecular phylogeny, deep reefs, submersible, Curaçao, Psilotris

## Abstract

We describe a new species of goby, *Varicus lacerta* **sp. n.**, which was collected from a mesophotic reef at Curacao, southern Caribbean. The new species is the tenth species of *Varicus*, all of which occur below traditional SCUBA depths in the wider Caribbean area. Its placement in the genus *Varicus* is supported by a molecular phylogenetic analysis of three nuclear genes and the mitochondrial gene cytochrome *b*. In addition, the new species has one anal-fin pterygiophore inserted anterior to the first haemal spine, which distinguishes *Varicus* species from most species in the closely related and morphologically similar genus *Psilotris*. *Varicus lacerta* **sp. n.** is distinguished from all other named species of *Varicus* by the absence of scales, having highly branched, feather-like pelvic-fin rays, and in its live coloration. We provide the cytochrome c oxidase I DNA barcode of the holotype and compare color patterns of all species of *Varicus* and *Psilotris* for which color photographs or illustrations are available. This study is one of several recent studies demonstrating the utility of manned submersibles in exploring the diversity of poorly studied but species-rich deep-reef habitats.

## Introduction

Operating out of Substation Curaçao (www.substation-curacao.com), the Smithsonian Institution’s Deep Reef Observation Project (DROP) uses the manned submersible *Curasub* to capture tropical marine fishes and invertebrates at depths up to 300 m, providing new information on the fauna that inhabits poorly studied deep-reef ecosystems. DROP’s exploratory submersible diving in the southern Caribbean has led to the discovery of a cache of undescribed fish biodiversity, some of which has been recently described (Van Tassell et al. 2012; Baldwin and Johnson 2013; Baldwin and Robertson 2013, 2014, 2015; Tornabene et al. 2016). Many of the new species belong to the Gobiidae, most notably the tribe Gobiosomatini (Van Tassell et al. 2012; Tornabene et al. 2016). This tribe comprises the American seven-spined gobies, a taxonomically and ecologically diverse clade of fishes that is endemic to the western Atlantic and eastern Pacific Oceans. A repeated pattern of rapid speciation via microhabitat specialization in this tribe has resulted in the Gobiosomatini becoming a model group for the study of adaptive radiation in the marine environment (Rüber et al. 2003; Taylor and Hellberg 2005; Tornabene et al. in press). One of the most ecologically and taxonomically diverse clades within the Gobiosomatini is the *Nes* subgroup, which comprises 39 species in 11 genera that inhabit a wide variety of marine habitats (Tornabene et al. 2016). Within the *Nes* subgroup, three genera have species described from mesophotic reefs below 50 m: *Pinnichthys* Gilmore, Van Tassell & Tornabene, 2016, with four species, all from deep reefs; *Psilotris* Ginsburg, 1953, with six species, one from deep reefs; and *Varicus* Robins & Böhlke, 1961 with nine species prior to this study, all from deep reefs (Tornabene et al. 2016). Here we describe a tenth deep-reef species of *Varicus* based on a single specimen that was collected at 129-147 m from Curaçao.

## Materials and methods

The new species was collected using the *Curasub* manned submersible. The sub has two hydraulic arms, one equipped with a suction hose and the other with a quinaldine-ejection system used to anaesthetize fishes. Specimens collected with the suction hose are deposited into a vented acrylic cylinder attached to the outside of the sub. The captured holotype was brought to the surface alive, where it was photographed and tissue sampled prior to fixation in 10% buffered formalin and subsequent storage in 75% ethanol.

Tissue from the holotype was stored in saturated salt-DMSO (dimethyl sulfoxide) buffer (Seutin et al. 1991). DNA extraction and cytochrome *c* oxidase subunit I (COI) DNA barcoding were performed as outlined by Weigt et al. (2012). To confirm the phylogenetic placement of the new species we also sequenced the mitochondrial gene cytochrome *b* and the nuclear genes Rag1, sreb2, and zic1. Following nomenclature of Chakrabarty et al. (2013), new sequences here constitute genseq-1 COI, cytb, Rag1, sreb2, and zic1. Primers and PCR conditions for amplifying these four loci were identical to those used in Agoretta et al. (2013). Sequences generated here were aligned with Gobiosomatini sequences from Tornabene et al. (2016) in *Geneious v. 9* (Biomatters, Ltd., Auckland). Substitution model choice and partitioning scheme were assessed using PartitionFinder (Lanfear et al. 2012). Phylogeny was inferred using Bayesian inference in the program MrBayes ver. 3.2, using two Metropolis-coupled Markov Chain Monte Carlo (MCMC) runs, each with four chains. The analysis was run for 10 million generations sampling trees and parameters every 1000 generations. Burn-in, convergence and mixing were assessed using Tracer (Rambaut and Drummond 2007) and by visually inspecting consensus trees from both runs.

All measurements were taken with digital calipers to the nearest 0.1 mm. Vertebral counts and pterygiophore patterns were taken from digital radiographs. Dorsal pterygiophore formula is that of Birdsong et al. (1988), and head pore terminology follows Akihito et al. (1988). Sensory papillae are described following the terminology of Sanzo (1911), with the exception that the interorbital series follows terminology described by Tornabene et al. (2016). All other morphological characters are as defined by Böhlke and Robins (1968) as modified by Van Tassell et al. (2012), who like many authors, differentiate the unsegmented spine from the segmented rays of the second dorsal, anal, and pelvic fins using the roman numeral ‘I’ for the spine followed by Arabic numbers for the soft rays. The holotype was deposited at the National Museum of Natural History, Smithsonian Institution
(USNM).

## Results

### Varicus lacerta sp. n.

Taxon classificationAnimaliaPerciformesGobiidae

http://zoobank.org/77FB8CDB-9B22-4F33-B76F-262C5606665F

**Figure 1.**
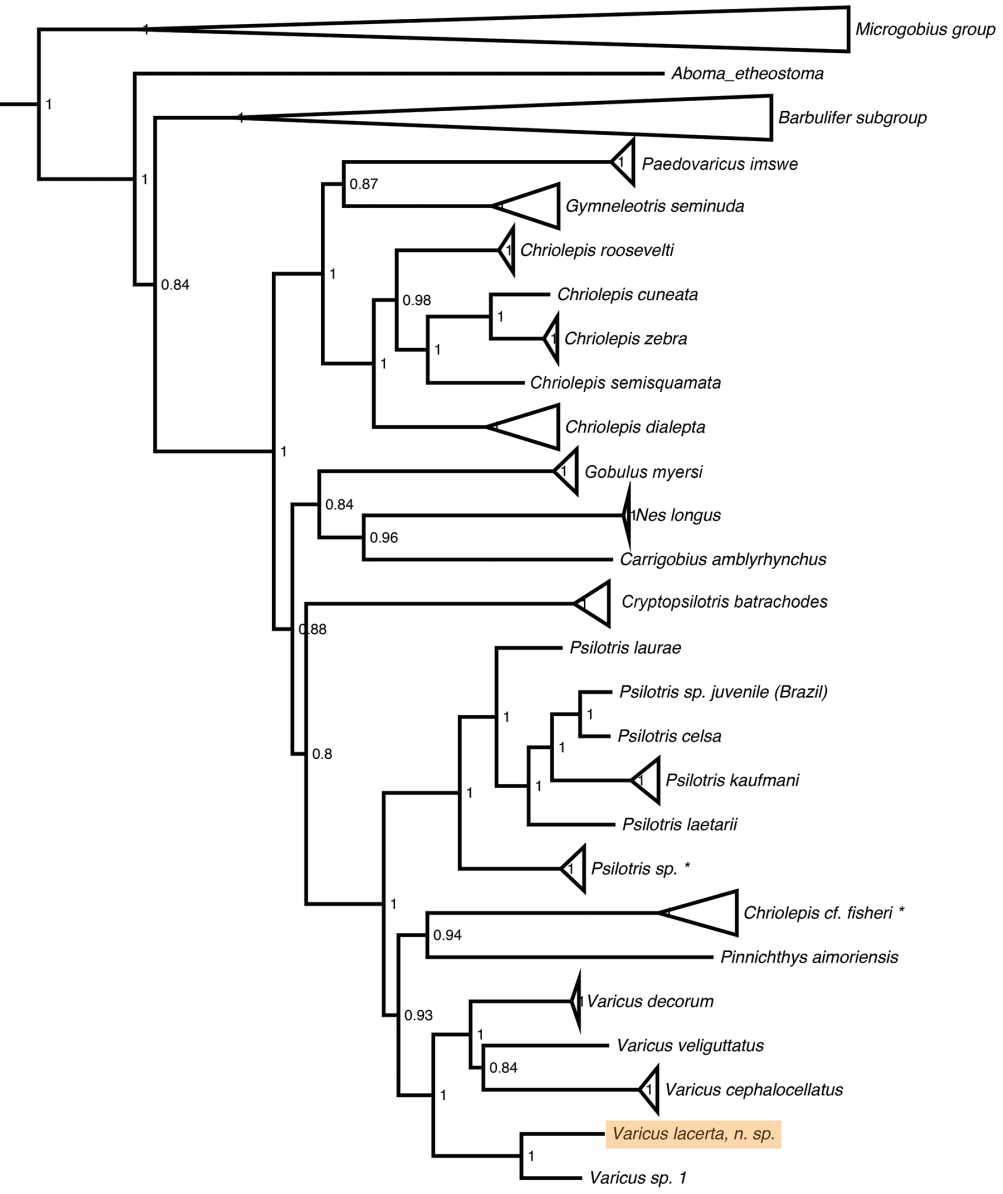
Molecular phylogeny of the Gobiosomatini based on three nuclear genes and one mitochondrial gene. Support values are Bayesian posterior probabilities. * indicates species that may be *Chriolepis fisheri*, see Tornabene et al. (2016) for more information.

**Figure 2.**
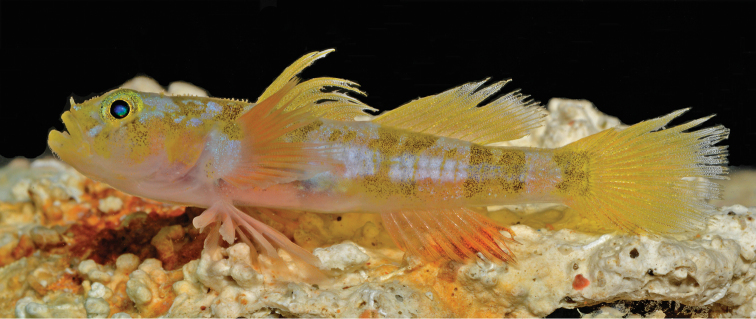
*Varicus lacerta* sp. n., holotype, USNM 434796, 36.2 mm SL, male, live. Photo by Barry Brown.

**Figure 3.**
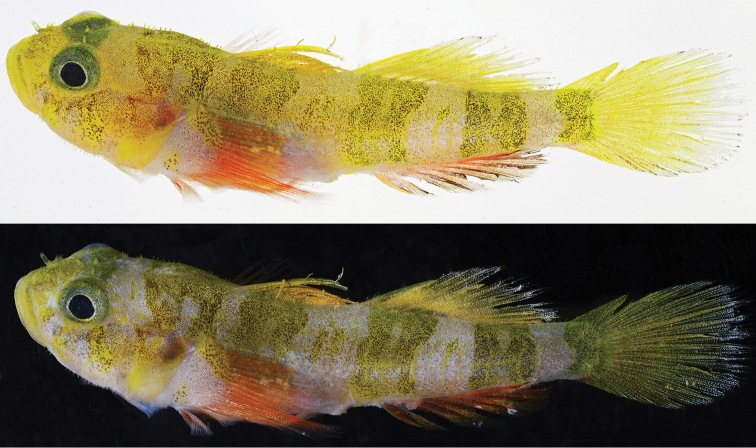
*Varicus lacerta* sp. n., holotype, USNM 434796, prior to preservation. Photos by Carole Baldwin and Ross Robertson.

**Figure 4.**
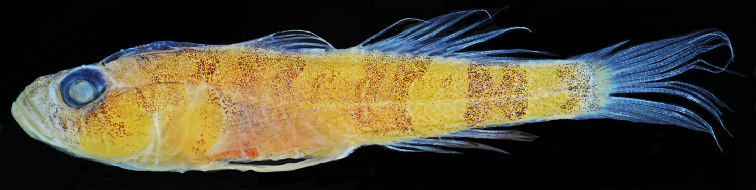
*Varicus lacerta* sp. n., holotype, USNM 434796, preserved. Photo by Sandra Raredon.

**Figure 5.**
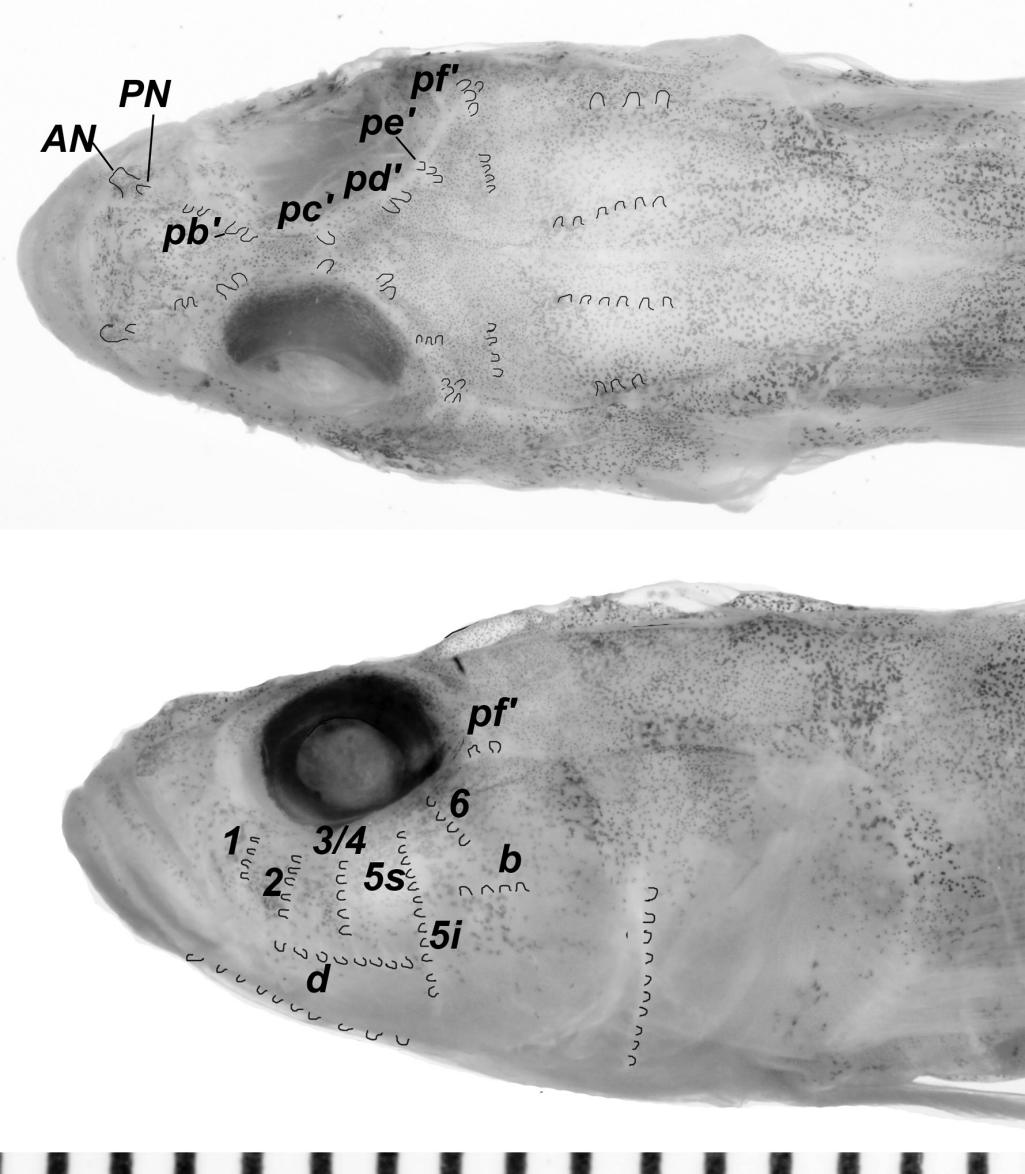
Sensory papillae pattern of *Varicus lacerta* sp. n. Scale-bar increments are millimeters. Photos by Sandra Raredon. Individual papillae are outlined in black for emphasis.

Figs 1 , 2 , 3 , 4 , 5

Varicus lacerta Godzilla Goby

#### Type locality.

Curaçao, southern Caribbean.

**Holotype.** USNM 434796, male, 36.2 mm SL, Curasub submersible, sta. CURASUB15-24, southern Caribbean, Curaçao, east of downline off Substation Curacao dock, near 12.083 N, 68.899 W, 129-143 m, quinaldine, 24 September 2015, Carole C. Baldwin, Darryl Felder, Bruce Brandt and Jennifer Felder.

#### DNA barcode of holotype.

ATAAAGATATTGGCACCCTCTATTTGATCTTCGGCGCCTGAGCTGGCATAGTCGGCACTGCTCTAAGCCTTCTTATTCGGGCAGAGCTAAGCCAACCTGGCGCCCTTTTAGGGGATGACCAGATCTACAACGTGATCGTTACTGCCCACGCCTTCGTAATAATCTTCTTTATAGTAATACCCGTCATGATTGGGGGCTTTGGGAACTGGCTCGTCCCTCTTATGATTGGGGCCCCCGATATGGCCTTTCCCCGAATAAATAACATAAGCTTCTGACTCCTCCCCCCCTCTTTCCTCCTGCTCTTAGCCTCCTCCGGCGTTGAAGCAGGCGCTGGCACAGGGTGAACCGTATACCCCCCCCTAGCCGGAAACCTCGCCCACGCCGGGGCCTCTGTTGATTTAACAATTTTTTCCCTCCACTTAGCAGGCATTTCCTCAATCCTAGGAGCCATTAACTTTATTACCACCATCCTCAACATAAAGCCCCCAGCAATCTCGCAATATCAAACCCCCCTTTTTGTATGGGCCGTGCTAATTACGGCTGTTCTTCTATTACTCTCCCTGCCCGTCCTAGCTGCAGGAATTACAATACTTCTTACCGATCGTAACCTAAATACAACCTTTTTTGACCCCGCAGGAGGGGGAGACCCCATTCTCTACCAACACCTCTTCTGATTCTT

#### Generic placement.

In addition to molecular characters supporting the phylogenetic placement (Fig. 1), the following morphological characters support the inclusion of the new species in *Varicus*: first dorsal spines VII; dorsal-fin pterygiophore formula 3-221110; vertebrae 11+16; hypurals 1-2 and 3-4 partially fused; one anal-fin pterygiophore inserted anterior to first haemal spine; anal-fin rays I,9 or fewer (I,7 in *Varicus lacerta*); head pores absent; transverse papillae rows 5i and 5s connected as a single continuous row; pelvic fins completely separate, lacking both anterior frenum and membrane connecting bases of innermost pelvic-fin rays; fifth pelvic-fin ray unbranched.

#### Diagnosis.

Second dorsal fin I,9; anal fin I,7; pectoral fin 18; no scales; cephalic papillae rows 5*s* and 5*i* connected, forming a single row; pelvic rays 1-4 highly branched and feather-like; one anal-fin pterygiophore inserted anterior to first haemal spine; body with five broad, indistinct, dark vertical bands washed with bright yellow in life; pelvic, pectoral and anal fins yellow-orange in life, dorsal, anal, and caudal fins yellow with faint orange tint.

#### Description.

General shape: body robust, widest and deepest at head, trunk tapering in width and depth posteriorly, dorsal head profile gradually sloping from dorsum to lips.

Median and paired fins: first dorsal fin VII, second spine longest, tips of spines projecting from fin membrane; second dorsal fin I,9, last ray branched to the base; anal fin I,7, last ray branched to the base; pectoral fin 18/18, fin extending posteriorly to vertical through anus; pelvic fins I,5, fins well separated, lacking both anterior frenum and membrane connecting bases of innermost rays; 4th pelvic-fin ray longest, extending posteriorly to anus; rays 1–4 connected by a thin membrane, each ray with one primary bifurcation followed by numerous thin branches off main branch that are united by a continuous membrane to the tip of the ray, giving each ray a feather-like appearance; 5th ray unbranched and 60–70% the length of 4th ray; caudal fin rounded, branched caudal-fin rays 15, segmented caudal-fin rays 17.

Squamation: no scales on head and trunk.

Head: jaw terminal, angled approximately 40 degrees from horizontal axis of body, extending posteriorly to a vertical at anterior end of pupil; anterior nares elongate narrow tubes; posterior nares inconspicuous openings covered by a short flap; no cephalic lateralis pores on head or preopercle; eyes large, dorsolateral, extending slightly above head profile; interorbital space narrow; operculum opening slightly wider than width of pectoral-fin base; teeth in upper jaw in two rows, outer row enlarged, canine-like, recurved, and evenly spaced, extending along most of premaxilla; inner rows smaller, more numerous, and more tightly spaced; teeth in lower jaw in three rows, outermost and innermost rows slightly enlarged, middle row smaller and more numerous; tongue truncate, tip with very slight indentation.

Morphometrics (% SL): head length 33.1; eye diameter 9.4; interorbital 2.6; snout length 8; upper-jaw length 12.4; predorsal length 40.1; body depth at origin of first dorsal 19.1; body depth at anal-fin origin 15.2; body depth at caudal peduncle 10.2; caudal-peduncle length 21.1; pectoral-fin length 24.0; pelvic-fin length 26.0; caudal-fin length 27.1.

Genitalia: male with short, conical, pointed papilla, wide at base and tapering distally to a point, no melanophores present; female unknown.

Color in life (Figs 2, 3): Ground color pale grey, head and body spangled with tiny silver and black dots, upper two-thirds of head and upper half of body with yellow tint that is more visible when fish photographed against white vs. black background (Figs 2 and 3, upper panel); breast, lower portions of head and opercle, chest, and lower portion of belly pale pinkish white.

Head with areas of bright yellow pigment heavily speckled with black dots on snout, along upper lip, as an irregular blotch over most of opercle, in a broad band across nape, and as two irregular bars below the eye, one beneath center of eye and extending to rear corner of mouth, the other running obliquely back from posteroventral corner of eye to lower corner of preopercle; iris greenish yellow, heavily speckled with silver and black dots; a thin silvery-white inner ring around pupil.

Body with four broad yellow bars heavily speckled with black dots, one on upper half of body under first dorsal fin; second and third extending from dorsal midline nearly to ventral midline, second positioned under anterior half of second dorsal fin and third under posterior corner of second dorsal and anterior half of caudal peduncle; fourth and narrowest bar covering most of posterior end of caudal peduncle and extending onto base of caudal fin; first three body bars (and bar across the nape) appearing as double bars due to irregular pale blotches in centers; interspaces between first three body bars with small, black-speckled yellow blotches and short, thin yellow bars; pale areas on head and trunk with silver, iridescent markings that are most conspicuous along mid-flank in the photograph of the live fish (Fig. 2)

First dorsal fin yellow with fine yellow and orange dots on the inner two-thirds of fin, gradually replaced with silvery white dots on membranes of outer one fourth of fin; second dorsal fin similarly colored, but with silvery speckling predominating on outer one-third of fin. Basal three-quarters of caudal fin yellow, spangled with orange (mainly) and whitish dots; outer one-quarter of fin with rays gray and membranes translucent with heavy silver-white speckling, rear edge of fin with darker grey pigment suffused with orange. Anal fin orange, strongly so distally in live fish and basally in freshly dead fish (Figs 2, 3, respectively); outer half of fin membranes heavily speckled with dark brown dots; fin rays with yellow tint distally. Pectoral-fin base white, heavily spangled with silver dots, a large, black-speckled yellow blotch on upper corner and a similar, smaller, more diffuse yellow blotch on lower corner; rays pink basally, orange-red speckled with silver centrally, fading to pink distally; sparse silver spangles scattered over fin. Pelvic fins pale, washed with pinkish-orange speckling.

Color in preservation (Fig. 4): Ground color yellowish pale, snout and mouth pale gray; various dark marks present in live fish visible in preserved fish as concentrations of dark brown dots: two indistinct short dark bars under eye: dark blotch on nape; four dark bars on body and at end of caudal peduncle; dark blotches at top and bottom corners of pectoral base.

Sensory papillae (Fig. 5): sensory papillae well developed, with notably elongate papillae on nape, snout, cheek, and ventral surface of head, giving head a hairy or spikey appearance (visible in Figs 2 and 3, less obvious in preservation); a series of 5 transverse papillae rows on side of head; transverse papillae rows 5i and 5s united as a single continuous row positioned anterior to row *b*, continuing ventrally below row *d*; interorbital papillae series well developed, each side of the interorbital possessing 2 *pb*’ papillae, 1 *pc*’ papilla, 2 *pd*’ papillae, 3 *pe*’ papillae, and a cluster of 3-4 *pf*’ papillae.

Vertebral skeleton: dorsal pterygiophore formula 3-221110; one anal-fin pterygiophore inserted anterior to first haemal spine; second neural spine expanded and slightly spatulate at tip; hypurals 1–2 fused with hypurals 3–4 along approximately one-half of their length; 27 vertebrae, 11 precaudal, 16 caudal.

#### Habitat.

The only known specimen was collected at 129–143 m. Quinaldine was dispersed around a yellow sponge (~20 cm tall) tentatively identified from videos by Allen Collins (National Marine Fisheries Service) as *Dactylocalyx pumiceus*, situated on a rocky outcropping along the deep-reef slope. After approximately 20 seconds the stunned fish emerged from a space in the rocky substrate at the base of the sponge and was captured. It is unclear whether the fish was originally in direct association with the sponge itself or was instead sheltering in spaces within the rock. Video of the capture taken from a high-definition video camera mounted on the outside of the *Curasub* is available online (https://youtu.be/UvxJEi-vER0). Subsequent collections targeting similar sponges and rocky substrates within this depth range at the type locality have not yielded additional specimens.

#### Distribution.

Known only from the type location in Curaçao.

#### Etymology.

The specific epithet ‘lacerta’ (Latin for ‘lizard’) is in reference to the reptilian or saurian appearance of this species, as indicated by its bright yellow and orange coloration, green eyes, disproportionately large head possessing raised ridges of papilla, and multiple rows of recurved canine teeth in each jaw. The common name Godzilla goby (gobio Godzilla in Spanish) refers to the radioactive reptilian monster from the sea that appeared in Japanese science-fiction films as Gojira, renamed Godzilla in subsequent English-language films.

## Discussion and comparisons

**Figure 6.**
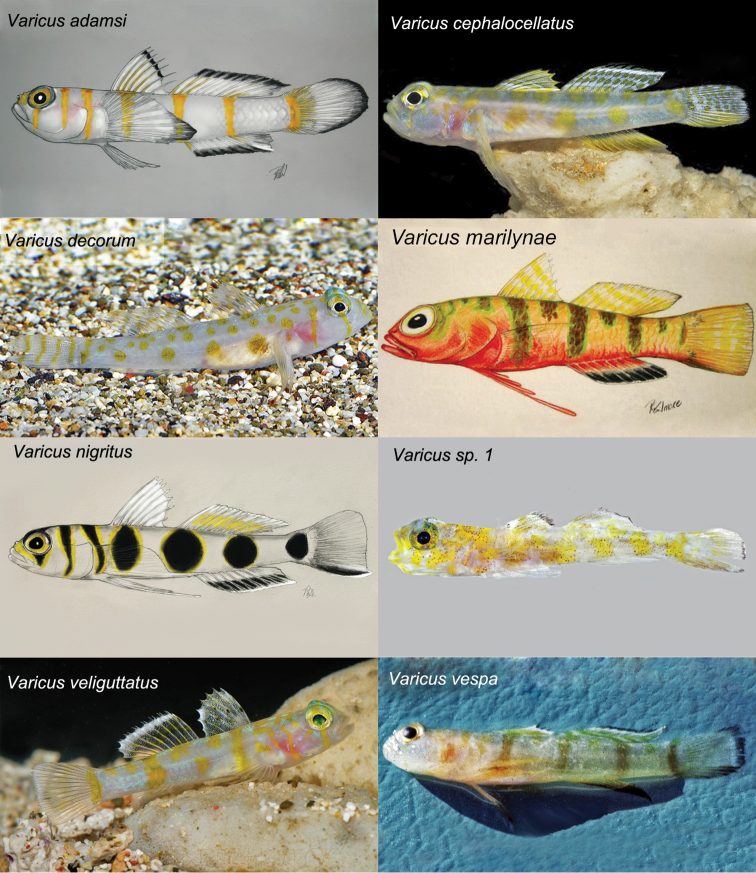
Coloration of species of *Varicus*. All illustrations by R. Grant Gilmore. Photographs by Barry Brown, Ross Robertson and Carole Baldwin (*Varicus* sp. 1), and the crew of the R/V Bellows (for *Varicus vespa*). Photos of *Varicus bucca* and *Varicus benthonis* not available.

The molecular phylogeny (Fig. 1) shows the new species nested within the genus *Varicus*, where it is recovered as sister to an undescribed species *Varicus* sp. 1 from Curaçao. This undescribed species is represented by a single specimen in poor condition that also lacks body scales, but is readily distinguishable from *Varicus lacerta* based on live coloration (see below). *Varicus lacerta* is easily distinguished from all described congeners by the absence of scales on the body and the presence of highly branched, feather-like pelvic-fin rays 1-4. *Varicus decorum* Van Tassell, Baldwin & Tornabene, 2016, lacks scales on most of the body, but possesses a pair of small, ctenoid scales on each side of the caudal peduncle near the base of the caudal fin, which are absent in *Varicus lacerta*. Live coloration also easily distinguishes *Varicus lacerta* from all other species of *Varicus* for which the live color pattern is known (Fig. 6). While *Varicus lacerta* has a color pattern of indistinct broad dark bars on a yellowish body, in five other *Varicus* species (*Varicus cephalocellatus* Gilmore, Van Tassell & Baldwin, 2016, *Varicus decorum*, *Varicus nigritus* Gilmore Van Tasell & Baldwin, 2016, *Varicus* sp. 1, and *Varicus veliguttatus* Van Tassell, Baldwin & Gilmore, 2016) the color pattern comprises blotches and spots of yellow or black on a white body. *Varicus lacerta* differs from *Varicus adamsi* Gilmore, Van Tassell & Tornabene, 2016, and *Varicus vespa* Hastings & Bortone (1981), in having indistinct broad dark bars on a yellowish body and yellow median fins vs narrow yellow bars on a white body and white median fins with black edges in *Varicus adamsi*, and narrow brown bars on a white body and white fins with black borders in *Varicus vespa*. While *Varicus lacerta* has a yellowish body with indistinct broad dark bars, uniformly yellow dorsal and caudal fins, and a yellow anal fin accentuated with dark orange, in *Varicus marilynae* Gilmore, 1979 the body is yellow above, reddish orange below, the body bars are narrow, green-edged and dark brown, the dorsal and tail fins have narrow yellow stripes and bars, and the anal fin is red with a black border.

The absence of scales and the presence of highly branched pelvic rays without fleshy tips make this species superficially similar to species of *Psilotris*. No single morphological character unambiguously distinguishes *Varicus* from *Psilotris*, but the most consistent morphological feature thus far is the presence of a single anal-fin pterygiophore inserted before the first haemal spine in *Varicus* versus two in all but one species of *Psilotris*. *Psilotris laurae* Van Tassell, Tornabene & Baldwin, 2016, has a single pterygiophore anterior to the haemal spine, and it is the only known deep-reef species of *Psilotris*. The relationship between anal-fin pterygiophore pattern and habitat depth warrants further investigation.

**Figure 7.**
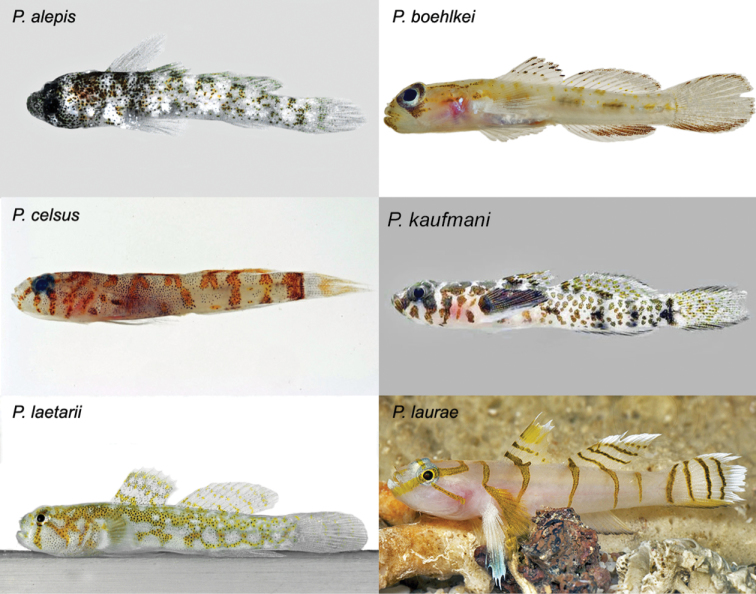
Coloration of species of *Psilotris*. Photos by Luiz Rocha, Jeffrey Williams, Ross Robertson, Barry Brown, and James Van Tassell.

Despite the morphological similarities between *Varicus lacerta* and species of *Psilotris*, the new species is easily distinguished by live coloration (Fig. 7). Only three members of *Psilotris* have discrete body bars: *Psilotris alepis* Ginsburg, 1953, with irregular grey bars on a white body speckled with black and brown; *Psilotris celsa* Böhlke, 1963, with irregular narrow orange bars on a white body and head; and *Psilotris laurae*, with narrow, strongly defined dark-yellow bars on a white head and body. *Psilotris boehlkei* Greenfield, 1993, *Psilotris kaufmani* Greenfield, Findley & Johnson, 1993, and *Psilotris laetarii* Van Tassell & Young, 2016, lack bars. *Varicus lacerta* can also be distinguished from *Psilotris boehlkei*, *Psilotris celsa*, *Psilotris kaufmani* and *Psilotris laurae* by having I,7 anal-fin rays (vs. > I,7; Table 1), and from *Psilotris alepis*, *Psilotris celsa*, and *Psilotris laetarii* in having 18 pectoral-fin rays (vs. <18; Table 1). The connection of papillae rows 5i and 5s further distinguishes *Varicus lacerta* from *Psilotris alepis* and *Psilotris boehlkei*, in which rows 5i and 5s are separate.

**Table 1.** Meristic and papillae characters for *Varicus* and *Psilotris*. AP = anal pterygiophores inserted anterior to haemal spine.

Species	Second dorsal	Anal	Pectoral	AP	Papillae rows 5i/5s	Body Scales	Basicaudal Scales
*Varicus adamsi*	I,9	I,7–8	18	1	connected	present	present
*Varicus benthonis*	I,8	I,7	16	1	separate	present	present
*Varicus bucca*	I,9	I,7–8	16–19	1	connected	present	present
*Varicus cephalocellatus*	I,10	I,9	19–20	1	variable	present	present
*Varicus decorum*	I,9	I,7–8	17	1	connected	absent	present
***Varicus lacerta* sp. n.**	**I,9**	**I,7**	**18**	**1**	**connected**	**absent**	**absent**
*Varicus marilynae*	I,8	I,7	16–18	1	connected	present	present
*Varicus nigritus*	I,9	I,8	17	1	connected	present	present
*Varicus veliguttatus*	I,8	I,6–7	17–19	1	connected	present	present
*Varicus vespa*	I,9	I,7 (rarely I,6 or I,8)	15–17	1	separate	present	present
*Psilotris alepis*	I,9 (rarely I,8)	I,7–8	15	2	separate	absent	absent
*Psilotris boehlkei*	I,9–10	I,9	16–18	2	separate	absent	absent
*Psilotris celsa*	I,9–10 (rarely I,8)	I,9–10 (rarely I,8)	16–17	2	connected	absent	absent
*Psilotris kaufmani*	I,10 (rarely I,9)	I,10 (rarely I,9)	16–19	2	connected	absent	absent
*Psilotris laetarii*	I,9–10	I,7–8	15–17	2	connected	absent	absent
*Psilotris laurae*	I,9	I,8	18	1	connected	absent	absent

## Supplementary Material

XML Treatment for Varicus lacerta
